# To a New Subscriber

**Published:** 1881-02

**Authors:** 


To a New Subscriber.—As an early answer is requested to your very proper questions, we would respectfully reply that the territory submerged by Noah's flood did not probably extend over an area of more than two thousand miles each way, or about the size of the state of Texas. The color of the sons of Noah was white or brunette, and there is no evidence of its ever having been changed in the case of either of them. Esau and Jacob were of the same race as the sons of Noah, or what is generally called the Semetic race. The ancient Egyptians styled them "yellow." Esau's covering of hair was simply a *lusus naturæ*, which his twin brother, Jacob, escaped.

